# Periodontal status and risk factors in patients with type 1 diabetes mellitus

**DOI:** 10.1007/s00784-024-06113-3

**Published:** 2025-02-05

**Authors:** Rosana Costa, Blanca Ríos-Carrasco, Paula López-Jarana, Cristina Cabral, Filipe Cunha, Maria Gonçalves, Marta Relvas

**Affiliations:** 1 Department of Medicine and Oral Surgery, University Institute of Health Sciences (IUCS-CESPU), Gandra, 4585-116 Portugal; 2 Department of Periodontology, School of Dentistry, Universidad de Sevilla, C/Avicena S/n, Sevilla, 41009 Spain; 3 Oral Pathology and Rehabilitation Research Unit (UNIPRO), University Institute of Health Sciences (IUCS- CESPU), Gandra, 4585-116 Portugal; 4 Endocrinology Department, Hospitalar Center of Tâmega e Sousa, Avenida do Hospital Padre Américo 210, Penafiel, 4560- 136 Portugal; 5 Associate Laboratory i4HB—Institute for Health and Bioeconomy, University Institute of Health Sciences— CESPU, Gandra, 4585-116 Portugal; 6 UCIBIO—Applied Molecular Biosciences Unit, Translational Toxicology Research Laboratory, University Institute of Health Sciences (1H-TOXRUN, IUCS—CESPU), Gandra, 4585-116 Portugal

**Keywords:** Type 1 Diabetes Mellitus, Periodontal disease, Periodontitis, Gingivitis

## Abstract

**Objective:**

Conduct a cross-sectional study to investigate the periodontal conditions and oral health behaviours among adult subjects with type one diabetes mellitus and compare them with those of a group of age- and gender-matched without diabetes. Furthermore, we also intend to evaluate the potential indicators of the risk for the development of periodontal disease.

**Methods:**

The evaluation was undertaken with patients with diabetes (*n* = 70) from a patients’ cohort of the the Hospitalar Center of Tâmega e Sousa and subjects without diabetes (*n* = 69).

**Results:**

The prevalence of periodontal disease showed significant differences between groups. Gingivitis reached a prevalence of 37.1% in patients with type one diabetes mellitus and periodontitis 55.7%. These systemically compromised patients exhibited a higher prevalence of Plaque Index, Bleeding on Probing and Periodontal Probing Depth and a reduced number of teeth when compared to the control group. The stage II was the most prevalent in the diabetes population, followed by the stage IV and most of diabetes subjects presented grade C progression.

**Conclusions:**

There is a higher prevalence of periodontal disease in type one diabetes mellitus individuals when compared to the controls. Age, Bleeding on Probing and number of cigarettes per day are associated with higher risk of periodontal disease in type one diabetes mellitus patients.

**Clinical relevance:**

Our study provides evidence about the prevalence of periodontal disease among type 1 diabetes mellitus and creates awareness regarding the factors that potentially contribute to worsening periodontal tissues. Furthermore, informing diabetic patients about the importance of early diagnosis and prevention of periodontal disease and the importance of reducing/quitting smoking.

## Introduction

Diabetes mellitus (DM) is a group of metabolic disorders characterized by hyperglycemia, resulting from definitive insulin function and/or reduced insulin production [1, 2]. The diagnosis of type 1 Diabetes mellitus (T1DM) is usually stablished at tender age, however recent epidemiological studies have shown that T1DM can appear also in adults and even at advanced age [3]. Among all the complications of diabetes such as dehydration and poor wound healing, we have also some diseases like heart and kidney failure, limb ischemia peripheral arterial disease, coronary disease, cerebrovascular disease, carotid disease and periodontal disease (PD) [1, 2].

Periodontal disease is a chronic inflammation of periodontal tissue associated with infection from Gram-negative bacteria contained in dental plaque, promoting a chronic and progressive local inflammatory response [4–8]. The progressive destruction of connective tissue and bone support can result in tooth loss and systemic inflammation [5]. The mechanism of interaction implies the possible induction of systemic inflammation from periodontal tissues, increasing the level of circulating inflammatory markers that pay an important role in the initiation and progression of PD [5, 9–13]. The amount of destruction of tooth-supporting tissues is measured in terms of clinical attachment level (CAL) and periodontal probing depth (PPD). Furthermore, bleeding on probing (BOP) has been associated to higher risk for attachment loss when compared with sites with no BOP [14].

The evidence suggests a two-way relationship, between PD and DM, with diabetes increasing the risk for periodontal disease and periodontal disease affecting glycemic control and increasing the severity of complications of diabetes. PD and DM are two chronic diseases that influence patient’s health and quality of life.The severity of both diseases was shown to be dependent on various risk factors such as metabolic control and duration of DM, as well as patient’s age, social behaviour, oral hygiene level and aggravating factors such as smoking. For that reason, DM patients should be advised that the cycle linking periodontal disease and diabetes mellitus can be managed for better oral and general health [15, 16].

A bidirectional relationship between type 2 Diabetes mellitus (T2DM) and periodontal disease has been documented in several studies, however significantly less studies have focused on the association between periodontal health and T1DM [16].

The aim of the study was to investigate the periodontal conditions and oral health behaviours among adult subjects with T1DM and compared them with those of a group of age- and gender-matched without diabetes. Furthermore, we also intend to evaluate the potential indicators of the risk for the development of periodontal disease.

## Materials and methods

### Study design

This single-center, cross-sectional study is reported according to STROBE guidelines [17]. The interventions were approved by the Ethical Committee of the Hospitalar Center of Tâmega e Sousa, Penafiel (reference: 22/2022) and performed according to the declaration of Helsinki.

Patients were carefully informed through oral and written explanations about the purpose and procedures of the study and an informed consent was obtained from all participants.

For inclusion, subjects had to be 18 years or more, had to be diagnosed with T1DM for at least 1 year and have at least 12 natural remaining teeth. Patients with history of systemic disease such as cancer, bone metabolic disease, HIV, history of radiotherapy or immunosuppressive/modulating therapy, as well as those who had taken antibiotics in the previous month, were excluded. The sample was selected using a non-probabilistic convenience sampling method. From October 2022 to July 2023, out of the 150 patients with T1DM approached at the Endocrinology Unit of the Hospitalar Center of Tâmega e Sousa, only 70 agreed to take part in the study. All the participants agreed to have dental examination for the purpose of the present study. Sixty-nine periodontally controls matched for age, gender and smoking habits were recruited.

### Medical visit

All patients with T1D attending the Endocrinology clinic, during their routine medical visit, were screened for eligibility. A complete diabetes medical history was collected from those who agreed to participate: diabetes duration, treatment, previous glycemic control, micro- and macrovascular complications, concomitant cardiovascular risk factors, blood pressure, height and body mass index (BMI/Kg/m2). The next appointment was then scheduled for a same day blood work (glycated hemoglobin (HbA1c)) and dental examination.

Good glycaemic control was defined as a glycated haemoglobin < 7% [18]. Insulin regimen was defined as follows: basal insulin (when only intermediate-acting or long-acting insulin was used); basal-plus (when in addition to the basal regimen an, rapid-acting insulin was used as correction insulin); basal-bolus (basal insulin plus a rapid-acting insulin as a prandial plus correction insulin); carbohydrate counting; and premixed insulin. Diabetic peripheral neuropathy was defined as loss of protective sensitivity tested by a 10 g monofilament or previous diagnosis. Diabetic nephropathy as considered the presence of an estimated glomerular filtration rate 60 mL/min/1.73m2 or and albumin-to-creatine 30 mg/g in a spot urine sample.

### Periodontal examination

Before periodontal examination, information regarding dental history was obtained by a questionnaire about the assessment of the patient’s oral health status.

Ten volunteers were used on two distinct days, separated by 48 h, to calibrate two examiners (RC, MR) who had previously received training from an experienced senior periodontic specialist in order to meet the measurement criteria. By measuring identical random volunteers, the two examiners were able to establish calibration and record the repeatability grade. For CAL and PD, the intra-examiner coefficients of correlation (CCI) were 0.97 and 0.98, while the inter-examiner CCIs were 0.99 and 0.98 respectively.The examiners were not blind concerning the diabetes status.

The following clinical parameters were recorded at six sites per tooth of each subject: plaque index (PI; O’Leary,1972 [19]), probing pocket depth using a manual probe (CP15), Clinical Attachment Level (CAL), bleeding on probing (BOP; Ainamo & Bay, 1975 [20]), gingival recession (REC), furcation involvement, and tooth mobility.

The staging and grading periodontitis was valued according to the new consensus report of the 2017 World Workshop on the Classification of Periodontal and Peri-Implant Diseases and Conditions [21] as follows: stage I, presence of interdental CAL at site of greatest loss 1 to 2 mm, radiographic bone loss at the coronal third (< 15%), no tooth loss due to periodontitis, maximum probing depth ≤ 4 mm mostly horizontal bone loss; stage II: presence of interdental CAL at site of greatest loss 3 to 4 mm, radiographic bone loss at the coronal third (15–33%), no tooth loss due to periodontitis, maximum probing depth ≤ 5 mm mostly horizontal bone loss; stage III: presence of interdental CAL at site of greatest loss ≥ 5 mm, radiographic bone loss extending to middle or apical third of the root, tooth loss due to periodontitis of ≤ 4 teeth, probing depth ≥ 6 mm, vertical bone loss ≥ 3 mm, furcation involvement class II or III and moderate ridge defect; stage IV: presence of interdental CAL at site of greatest loss ≥ 5 mm, radiographic bone loss extending to middle or apical third of the root, tooth loss due to periodontitis of ≥ 5 teeth, masticatory dysfunction, secondary occlusal trauma (tooth mobility degree ≥ 2), bite collapse, drifting, flaring and less than 20 remaining teeth (10 opposing pairs).

The grade of periodontitis was valued according to the indirect evidence of progression based in the percentage of bone loss/age and case phenotype and risk factors (smoking habits and HbA1c levels), as follows: Grade A (slow rate of progression), Grade B (moderate rate of progression) and Grade C (rapid rate of progression).

### Statistical analysis

Data analysis was carried out using the IBM SPSS (Statistical Program for Social Sciences), version 29.0 for Windows. Descriptive statistics were expressed as means and standard deviations for quantitative variables and as frequencies and percentages for qualitative variables. Clinical data were initially examined for normality with the Shapiro-Wilk test. The chi-square test was used to evaluate the association between qualitative variables (gender, age groups, smoking, and oral hygiene habits) between the two groups under study. The independent “t” test was used to compare periodontal indices between the two groups. To measure the magnitude of the effect, Cohen’s d was used with the following guidelines:| d| ≤ 0.20 expected as a small effect,| d| = 0.50 as a moderate effect and| d| ≥ 0.80 as a large effect (Cohen, 1988). The Mann-Whitney test was used to compare periodontal indices between controls and T1DM patients, in different age groups (19–30 years; 31–49 years and ≥ 50 years). To estimate the factors associated with periodontitis, binary logistic regression models were performed, using the Backward method. The significance level used was *p* <.05.

## Results

One hundred and thirty-nine individuals were selected for the study according to the inclusion and exclusion criteria. Among the individuals analyzed, 69 controls and 70 T1DM patients were included. The demographic characteristics of the population are presented in Table 1. Patients with T1D and controls were well matched for age, gender and smoking status. Concerning the smoking habits, the proportion of active smokers is very similar between controls (20.3%) and T1DM subjects (18.6%), with 14 of the 27 smokers (51.8%) smoking less than 10 cigarettes per day and 13 (48.2%) 10 or more cigarettes per day.

**Table 1:** Age, gender and smoking habit in controls and type 1 diabetes mellitus patients

	Controls (*n* = 69)	T1DM (*n* = 70)	χ^2^	*p*
**Gender**				
Male	40 (58.0)	41 (58.6)	0.05	0.943
Female	29 (42.0)	29 (41.4)		
mean ± SD	34.6 ± 13.0	35.2 ± 13.6		
**Age**				
19–30 years	33 (47.8)	34 (48.6)	0.65	0.722
31–49 years ≥ 50 years	28 (40.6) 8 (11.6)	25 (35.7) 11 (15.7)		
**Smoking Habits**				
Non-Smoker	55 (79.7)	57 (81.4)	0.066	0.798
Smoker	14 (20.3)	13 (18.6)		
< 10 cigarettes	6 (42.9)	8 (61.5)	0.942	0.332
≥ 10 cigarettes	8 (57.1)	5 (38.5)		

p- level of significance; SD-standard deviation; T1DM- Type 1 Diabetes Mellitus; X^2^-The chi-square test

Note: Values indicate number of subjects and percentages

Table 2 summarizes the main characteristics of T1DM population. The average since the diagnosis of diabetes was 2.8 years (± 1.12), and the mean body mass index, glucose level and HbA1c were 25.21 (± 3.68), 183.91 (± 86.07) and 7.72 (± 1.47), respectively. Forty-five (64.3%) of T1DM individuals were poorly controlled. Most individuals (70.0%) do not use an insulin infusion pump and, regarding the insulin regimen, the majority (61.4%) use carbohydrate counting. Concerning the possible complications normally associated with diabetes, it was found that the retinopathy (24.3%), was the prevailing chronic diabetic complication, followed by nephropathy (14.3%), diabetic foot (14.3%) and neuropathy (10.0%).

**Table 2:** Medical characteristics of type 1 diabetes patients

		Value
**Duration of diabetes (years)**,** mean ± SD**		2.8 (1.1)
**BMI (Kg/m2)**,** mean ± SD**		25.21 (3.7)
**Glycated haemoglobin**,** n (%)**		
Well Controlled < 7%		25 (35.7)
Poorly Controlled		45 (64.3)
**Insulin infusion pump**,** n (%)**		
Yes		21 (30.0)
No		49 (70.0)
**Insulin Regimen**,** n (%)**		
Basal		1 (1.4)
Basal + bolus		24 (34.3)
Basal plus		1 (1.4)
Carbohydrate counting		43 (61.4)
Pre-mixed		1 (1.4)
**Arterial hypertension**,** n (%)**		14 (20.0)
**Chronic diabetes complications**,** n(%)**		
**Number of complications**,** mean ± SD**		0.96 (1.7)
**Microvascular complications**,** n (%)**		
Retinopathy		17 (24.3)
Nephropathy		10 (14.3)
Neuropathy		7 (10.0)
Diabetic foot		10 (14.3)
**Macrovascular complications**,** n (%)**		
Peripheral arterial disease		3 (4.3)
Coronary Disease		2 (2.9)
Amputation		2 (2.9)
Cerebrovascular disease		1 (1.4)
Carotid disease		1 (1.4)

n- number of individuals in the sample; BMI- body mass index; SD- standard deviation; T1DM- Type 1 diabetes mellitus

Table 3 shows the oral health behavior of the study population. Most individuals reported brushing their teeth twice a day (70 individuals), followed by those who reported having this habit three times a day (52 individuals), with the highest percentage of individuals brushing their teeth 3 times a day belongs to the control group (55.1%), with a statistically significant relationship (χ^2^ (3) = 24.94; *p* <.001). There was also a statistically significant relationship between the use of oral hygiene aids and the group they belong to (χ^2^ (2) = 9.02; *p* =.003), out of 57 individuals using interdental brushes/flossing, 37 (64.9%) belongs to the control group.

**Table 3:** Oral health habits in controls and type 1 diabetes patients

	Controls (*n* = 69)	T1DM (*n* = 70)	χ^2^	*p*
**Oral hygiene habits**				
Yes	69 (100)	66 (94.3)		
No	0 (0.0)	4 (5.7)		
**Frequency of tooth brushing**				
1 x day	0 (0.0)	11 (16.7)		
2 x day	31 (44.9)	39 (59.1)		
3 x day	38 (55.1)	14 (21.2)	24.94	< 0.001
> 3 x day	0 (0.0)	2 (3.0)		
**Interdental brushes/flossing**				
Yes	37 (53.6)	20 (28.6)		
No	32 (46.4)	50 (71.4)	9.02	0.003

p- Level of significance; X^2^- The chi-square test; n- number of individuals in the sample; T1DM- Type 1 Diabetes Mellitus

Regarding the diagnosis of periodontal disease, there were statistically significant differences between the groups (χ^2^ (2) = 49.66; *p* <.001). As shown in Tables 4 and 63.8% of the controls and 7.1% of T1DM individuals had periodontal health, gingivitis was present in 18.8% of the controls and 37.1% of T1DM subjects, with periodontitis reaching a prevalence of 17.4% in controls and 55.7% in T1DM patients. Out of the 12 controls with periodontitis, 6 (50.0%) had a diagnosis of stage I, 5 (41.7%) had stage II and 1 (8.3%) had stage III. Concerning the 39 diabetes subjects with periodontitis, 8 (20.51%) had stage I, 14 (35.9%) had stage II, 6 (15.4%) had stage III and 11 (28.2%) had stage IV. In which concerns the extent of periodontitis, regardless of the stage, the majority have presented generalized periodontitis. In relation to the progression of periodontitis, there were statistically significant differences between the groups (χ^2^ (2) = 30.23; *p* <.001), out of the 12 controls with periodontitis, 7 (58.4%) were classified as grade A, 4 (33.3%) with grade B and 1 (8.3%) with grade C. Out of the 39 diabetes patients, 9 (23.1%) were classified as grade B and 30 (76.9%) with grade C.

**Table 4:** Periodontal status of the studied population

	Controls *n* (%)	T1DM *n* (%)	χ^2^	*p*
**Healthy**	44 (63.8)	5 (7.1)	49.66	
**Gingivitis**	13 (18.8)	26 (37.1)		
Generalized Localized	8 (61.5) 5 (38.5)	26 (100) 0		< 0.001
**Periodontitis**	12 (17.4)	39 (55.7)		
Stage I	6 (50.0)	8 (20.51)	6.71 0.082	
Generalized Localized	2 (33.3) 4 (66.7)	5 (62.5) 3 (37.5)		
Stage II	5 (41.7)	14 (35.9)		
Generalized Localized	3 (60.0) 2 (40.0)	12 (85.7) 2 (14.3)		
Stage III	1 (8.3%)	6 (15.4)		
Generalized Localized	0 1 (100)	6 (100) 0		
Stage IV	0	11 (28.2)		
Generalized Localized		11 (100) 0		
Grade A Grade B Grade C	7 (58.4) 4 (33.3) 1 (8.3)	0 9 (23.1) 30 (76.9)	30.23	< 0.001

p- Level of significance; X^2^- The chi-square test; n- number of individuals in the sample; T1DM- Type 1 Diabetes Mellitus

### Comparison of periodontal parameters (PI, BOP, CAL and PPD) and number of teeth between the two groups

The clinical characterization of periodontal disease includes several measurements that indicate the development of the disease. The most relevant determinations are PI, BOP, PPD, CAL and the number of teeth. T1DM subjects shown a significant increase in PI, BOP, PPD and CAL levels compared to controls (*p* <.001 for all parameters). On the other hand, the number of teeth is significantly lower in patients with T1DM compared to controls (*p* <.001). (Table 5).

**Table 5:** Comparison of periodontal parameters and the number of teeth in controls and type 1 diabetes patients

Periodontal parameters	Controls (*n* = 69)	T1DM (*n* = 70)	t	Cohen’s d	*p*
**PI**	23.38 ± 19.78	48.13 ± 26.67	6.21	1.05	< 0.001
**BOP**	10.32 ± 10.94	32.02 ± 18.00	8.57	1.45	< 0.001
**PPD**	1.81 ± 0.59	2.57 ± 0.84	6.20	1.04	< 0.001
**CAL**	1.23 ± 1.37	2.44 ± 1.63	4.71	0.80	< 0.001
**Number of Teeth**	28.35 ± 2.48	25.89 ± 5.28	3.51	0.60	< 0.001

n- number of individuals in the sample; PI- Plaque Index; BOP- Bleeding on Probing; CAL- clinical attachment level; Cohen’s d - effet size; p - level of significance; PPD- periodontal probing depth; t-statistic derived from de independent t- test; T1DM- Type 1 Diabetes Mellitus; Data summarized as mean and standard deviation

### Comparison of periodontal parameters between controls and diabetes patients, according to different age groups

We compared periodontal parameters between controls and type 1 diabetes patients, in different age groups. Table 6 shows that in the age groups from 19 to 30 years old, from 31 to 49 as well as ≥ 50 years old, the T1DM subjects presented significantly higher values in terms of PI, BOP, PPD and CAL compared to the controls. In the age group of 50 years or more, diabetes patients present significantly higher values in relation to the CAL level (*p* =.014).

**Table 6:** Comparison of periodontal parameters between type 1 diabetes patients and controls, according to different age groups

Periodontal parameters	Controls (*n* = 33)	T1DM (*n* = 34)	*p*	Controls (*n* = 28)	T1DM (*n* = 25)	*p*	Controls (*n* = 8)	T1DM (*n* = 11)	*p*
	*19–30 years*			31-49 *years*			*≥ 50 years*		
**PI**	13 [9.7; 34.0]	35.8 [23.9; 52.1]	< 0.001	13.5 [8.7; 28.0]	50.0 [31.3; 71.0]	< 0.001	45.0 [28.1; 57.5]	57.5 [41.3; 90.3]	0.160
**BOP**	6.5 [3.0; 10.5]	27.3 [17.6; 37.5]	< 0.001	4.5 [2.0; 9.3]	29.9 [24.4; 44.6]	< 0.001	29.4 [14.6; 37.5]	38.0 [18.5; 55.0]	0.283
**PPD Total**	1.5 [1.3; 1.8]	2.3 [1.9; 2.6]	< 0.001	1.8 [1.4; 2.1]	2.5 [2.2; 3.0]	< 0.001	3.1 [2.0; 3.5]	2.7 [2.6; 4.2]	0.457
**CAL Total**	0 [0; 2.1]	2.0 [0.0; 2.2]	0.001	2.0 [0.0; 2.7]	2.6 [2.2; 3.5]	0.002	2.0 [0.5; 3.1]	3.5 [2.6; 4.5]	0.014
**Number of Teeth**	28 [28; 28]	28 [28; 30.25]	0.426	28 [28; 30]	26 [23; 28]	< 0.001	26 [21.5; 28]	20 [16; 23]	0.018

PI- Plaque Index; BOP- Bleeding on Probing; CAL- clinical attachment level; n- number of individuals in the sample; PPD- periodontal probing depth; T1DM- Type 1 Diabetes Mellitus; p value derived from the Mann-Whitney test; data summarized as median and interquartile range

Predictors of periodontal disease in patients with T1D were studied using a multivariate logistic regression analysis. We included the variables with a potential relationship to the presence of periodontal disease (Table 7). In patients with T1DM, age, BOP, and number of cigarettes per day were independently associated with periodontal disease with an odds ratio (OD) of 1.08 (per 10-year increased in age), 1.08, and 1.45, respectively. The obtained model has an accuracy of 77.1%, a sensitivity of 71.0% and a specificity of 81.2%.

**Table 7:** Binary logistic regression analysis to estimate the association between different variables and periodontitis among type 1 diabetes patients

	Inicial Model					Final Model				
Variables	B	Standard Error	*p*	OR	95% CI	B	Standard Error	*p*	OR	95% CI
**Age**	0.058	0.034	0.086	1.06	[0.99; 1.13]	0.074	0.025	0.003	1.08	[1.03; 1.13]
**BOP**	0.10	0.032	0.002*	1.11	[1.04; 1.17]	0.081	0.026	0.002	1.08	[1.03; 1.14]
**PI**	0.004	0.016	0.815	1.00	[0.97; 1.03]					
**Frequency of tooth brushing**	-0.182	0.500	0.715	0.83	[0.32; 2.22]					
**Nº cigarettes/day**	0.451	0.222	0.042*	1.57	[1.02; 2.42]	0.371	0.159	0.02	1.45	[1.06; 1.98]
**Disease duration**	-0.392	0.385	0.309	0.68	[0.32; 1.43]					
**Number of complications**	0.453	0.347	0.192	1.57	[0.80; 3.10]					
**HbA1c**	-0.457	0.285	0.109	0.63	[0.36; 1.10]					
**Constant**	-0.882	2.449	0.719	0.414		-5.26	1.36	< 0.001		

BOP- Bleeding on Probing; B- estimates for the slope coefficients of the univariate logistic regression model containing only this variable; p- value associated with the statistical coefficient test; S.E- estimated standard error for the estimated coefficient; CI- confidence interval of 95% for odds ratio; HbA1c- glycated haemoglobin; OR- estimated odds ratio; p- level of significance; PI- Plaque Index

## Discussion

This cross-sectional study is the first population-based cohort study in Portugal to assess patients with T1DM in terms of prevalence and the potential risk of developing PD.

The main objective of this study was to investigate the periodontal conditions and oral health behaviours among adult subjects with T1DM and compare them with those of a group of age- and gender-matched without diabetes and to evaluate the potential indicators of the risk for the development of periodontal disease.

According to our results, there was an increased prevalence of diabetes in males when compared to females. These findings are in agreement with the last Annual report of the National Diabetes Observatory 2023 [22].

In the present study, gingivitis and periodontitis reached a higher prevalence in T1DM group when compared to the control group. Our results are consistent with other studies showing that the prevalence and severity of periodontal disease are higher in T1DM patients in comparison with healthy individuals [16, 23].

In our survey, the prevalence of periodontitis-stage I, stage II, stage III, or stage IV- showed statistical differences between the T1DM patients and the control group. Although, we found that T1DM subjects had a higher prevalence of stages II and IV, while controls had a higher incidence of stages I and II.

The more advanced stages of periodontitis (III and IV) were more prevalent in the type 1 diabetes group compared to the control group. So far, we have not found any study that evaluates the severity of periodontitis by stage according to the current classification to compare our results. However, Roy et al. [16]. compared T1DM patients with controls classifying periodontitis into (mild, moderate, and severe) and found no differences between groups.

Regarding the extent of periodontal diseases, there was a higher prevalence of generalized extension in T1DM subjects. The progression rate of PD did differ between T1DM individuals and controls. Most of type 1 diabetes subjects presented grade C, which indicate a rapid rate progression of PD. According to the new consensus report of the 2017 World Workshop on the Classification of Periodontal and Peri-Implant Diseases and Conditions [21], these results show an association with the poor glycemic control that is seen in most of T1DM patients and with the smoking habits. To date, there are also none studies of disease progression according to the current classification in type 1 diabetes.

The increased glucose levels in salivary and gingival crevicular fluid, as a result of hyperglycaemia, directly promote periodontopathic bacteria proliferation and inflammation [23]. Bleeding on probing showed significantly higher values in T1DM individuals, which suggests greater susceptibility for PD. These observations were in agreement with all of the studies in diabetes showing that type 1 diabetes patients have greater periodontal inflammation in response reaction to microbial plaque accumulation [24–26]. The role of microbial plaque in the etiology of periodontal disease is uncontested. A much less pronounced relationship between dental plaque and severe periodontitis have been demonstrated [27]. In the present study, although there were no significant differences in terms of oral hygiene habits, the T1DM group showed higher levels of plaque accumulation compared to the controls. As a sign of inadequate oral hygiene habits is the non-use of brushing aids in 71.4% of type 1 diabetes individuals, which predisposes the patient to a greater accumulation of interdental biofilm and consequently greater gingival inflammation. A study carried out by Bourgeois et al. [28] suggests a positive impact of interdental aids in reducing interproximal bleeding. From week one, the observed reduction was 47% and at 3 months, bleeding was reduced by 71% compared to those who used only manually toothbrushing.

The presence of chronic inflammation promotes the destruction of periodontal tissues, which can lead to tooth loss. According to our results, T1DM individuals showed deeper pathological pockets and higher CAL when compared to controls. These periodontal parameters are associated with the amount of destruction of tooth-supporting tissues that could lead to tooth loss. The results showed that the number of teeth was significantly lower in T1DM patients, in accordance with the study carried out by Poplawska-Kita et al. [29] in which T1DM individuals, especially those poorly controlled had also lower number of teeth. However, in the analyzed studies, few or almost none evaluated this parameter.

In the present study, comparing these periodontal parameters according to age group, we can conclude that at the younger ages of 19–30 years and 31–49 years, T1DM individuals showed a greater accumulation of bacterial plaque, which predisposes to greater gingival inflammation and consequently the presence of periodontal probing depth, compared to individuals in the control group. If nothing is done to reduce this inflammatory condition, periodontal destruction will occur, which can lead to tooth loss that is usually seen in older ages. Regarding the results, older patients showed more significant tooth loss, which suggests that the expression of periodontal disease increases with age. These findings suggest that oral health education, diagnosis and treatment of periodontal disease should be recommended as early as possible by health professionals, especially in T1DM individuals.

Some studies indicate that bacterial plaque by itself is a poor predictor of subsequent periodontal disease activity, with several additional risk factors playing a role in its pathogenesis. The elevated inflammatory state in diabetes favour to both microvascular and macrovascular complications [16, 17]. It is amply documented that people with diabetes mellitus have higher risk of periodontal disease, and periodontal disease has been considered as the six complication of diabetes [2, 8, 30]. In the current study, among the complications of diabetes, the retinopathy (24.3%) was the most prevalent, followed by nephropathy (14.3%), diabetic foot (14.3%) and neuropathy (10.0%). According to the results obtained by Roy et al. [16] the most prevalent complication was retinopathy (22%) followed by nephropathy (14%) which is in line with our results. The mechanism by which diabetes influences the periodontal tissues are stated to be similar to the pathogenesis of the micro and macrovascular complications of diabetes mellitus.

The evidence suggests that the duration of diabetes, poor metabolic control, and the presence of diabetes complications are important aspects to consider in the evaluation of diabetes as a risk factor for periodontal disease [17, 24, 25]. According to a study carried by Relvas et al. [31], the risk of periodontitis is increased by approximately eightfold in patients with diabetes as compared with patients without DM. However, other authors reported no significant differences in the Community Periodontal Index (CPI) between groups with or without diabetes [32].

The HbA1c level provides a reliable index for measurement of glycaemic control in individuals with DM. Several studies suggests that the glycaemic control is of key importance in determining increased risk of periodontal disease [29]. This hypothesis was based on findings of Dicembrini et al. [5] and Jensen et al. [33] who reported a significant correlation between the prevalence of PD and glycaemic control, which increased severity of early biomarkers of periodontal individuals. Pro-inflammatory cytokines are elevated in diabetes, which can increase the connective tissue damage and impair wound healing [10, 12, 13, 34]. According to our findings, out of 70 T1DM individuals, 64.3% were poorly controlled.

Researchers find smoking to be a risk factor for periodontal disease; the smoker suffer from more severe forms of periodontitis and sowed more resistance to periodontal therapy [35]. A study carried out by Linden et al. [36] in Northern Ireland aimed to investigate the relationship between cigarette smoking and periodontal destruction in young adults. Twenty-four per cent of the smokers showed higher deep pocketing or loss of periodontal attachment (≥ 6 mm) compared with five per cent of the non-smokers.

According to the predictive model, in T1DM group, the variables identified as determinants of periodontal disease were age, BOP and number of cigarettes per day. Periodontitis showed a relationship also with the number of cigarettes per day increasing the risk by about 1.45 times in T1DM subjects.

It would be important to expand the sample of the diabetic population by including more hospital centers in Portugal, in order to generalize these findings to other groups of people with diabetes, as this is a single-centre study. In addition, longitudinal follow-up should be developed to explore the long-term impact of DM1 on oral health. In addition, studies are needed to make the comparison with our results as reliable as possible.

## Conclusion

Our results showed a higher prevalence of periodontal disease in T1DM individuals when compared to the controls. Furthermore, T1DM patients presented more severe forms of periodontal disease and faster degrees of progression compared to those without diabetes. T1DM subjects showed higher periodontal parameters when compared to the control group, which could lead to the destruction of tooth-supporting tissues and consequent tooth loss.

In addition, it is important to consider the risk factors such as age, BOP and number of cigarettes per day, which seems to predispose patients even more to periodontal destruction in systemically compromised individuals.

## Data Availability

No datasets were generated or analysed during the current study.
